# A Safe and Multifunctional γ-PGA Hydrogel Platform: Endotoxin-Controlled Injectable Fillers and Antimicrobial Wound Dressings

**DOI:** 10.3390/molecules30214205

**Published:** 2025-10-28

**Authors:** Bingbing Wang, Zejing Chu, Jingyu Wei, Ruixiang Mai, Yuan Wang, Xiaocui Wang, Yi Hou, Na Zheng, Jiao Sun, Biao Dong

**Affiliations:** 1 College of Food and Drug Engineering, Jilin Provincial Economic Management Cadre College, Changchun 130015, China; 2 College of Electronic Science and Engineering, Jilin University, Changchun 130012, China; 3 College of Food Science and Engineering, Jilin University, Changchun 130062, China; 4 Jilin Folialux Bio-Tech Co., Ltd., Changchun 130051, China; 5 College of Basic Medical Sciences, Jilin University, Changchun 130061, China

**Keywords:** γ-polyglutamic acid, γ-PGA, injectable micelle, antibacterial wound dressing

## Abstract

In response to the limitations of hyaluronic acid (HA)—such as its high cost, short durability, and instability—in anti-aging aesthetic applications, this study developed a novel injectable micelle system, with a triple network structure. It is the particle size of approximately 400 nm and the elevated potential that enhance the crosslinking density and mechanical strength of the hydrogel. Importantly, following ultrafiltration and purification processes, the material’s hemolysis rate measured by spectrophotometry was only 3.25%, and endotoxin levels measured by the LAL assay were less than 0.5 EU/mL (test conditions: 37 °C, pH = 7, detection limit: 0.125 EU/mL). Building on this safe and stable material platform, we further designed an antibacterial wound dressing by functionalizing γ-PGA with penicillin or benzalkonium chloride. It reduced the cellular activity of Staphylococcus aureus by 78.9% and 84.2%, respectively. The outstanding safety profile, combined with customizable functionality, positions this γ-PGA-based platform as a promising multifunctional biomaterial meeting practical standards for both aesthetic medicine and wound care applications.

## 1. Introduction

Skin aging is a biological phenomenon that the body inevitably undergoes during the natural physiological process. As we age, the ability of keratinocytes to synthesize moisturizing factors such as amino acids and urea diminishes, preventing the intercellular spaces of the stratum corneum from being adequately filled. This leads to a loosening of the barrier structure, directly resulting in a continuous increase in transepidermal water loss (TEWL): Individuals under 20 years old generally exhibit lower TEWL values, with most falling within the range of 8–10 g/(m^2^·h). Even when TEWL temporarily rises to around 12 g/(m^2^·h) due to brief stressors (such as staying up late or mild sunburn), the skin can rapidly activate metabolic mechanisms to repair barrier damage and return to normal levels within a short period. As age increases from 20 to 40 years old, TEWL values gradually rise from 10 g/(m^2^·h) to 20 g/(m^2^·h). Compared to earlier ages, TEWL values nearly double in individuals over 40 years old. Persistently elevated TEWL levels disrupt cellular metabolism, leading to a significant increase in free radicals produced by mitochondria. Excessive free radicals attack collagen, elastic fibers, and cellular DNA [1]. When this damage exceeds the body’s repair capacity, it can lead to skin pigmentation or spots [2]. When aging skin is injured, it can result in the formation of a wound that may remain unhealed for months or even years [3]. At the same time, it is often accompanied by issues such as bacterial infections. The most common pathogenic bacteria, Staphylococcus aureus, not only produces multiple toxins that destroy skin tissue but also resists phagocytic clearance, exacerbating wound inflammation, suppuration, and even triggering systemic infections. Additionally, wounds may harbor mixed infections involving Pseudomonas aeruginosa, Staphylococcus epidermidis, and other pathogens. Therefore, it is an extremely important method in the medical aesthetics industry to artificially replenish skin moisture and delay skin aging. Currently, the main material used for filling and hydration in the medical aesthetics industry is hyaluronic acid, also known as hyaluronan. As a hydrating material injected into the dermal layer, it has good biocompatibility and plasticity. However, its high cost, short duration, unstable filling strength, and potential for causing allergies limit its use [4].

Gamma-polyglutamic acid (γ-PGA) is a homogeneous polypeptide formed by the polymerization of left- and right-handed optically active glutamic acid monomers through amide bonds at the gamma position [5]. It has many advantages, such as good biocompatibility, biodegradability, moisturizing properties, and antibacterial properties [6]. Therefore, γ-PGA, as a biosynthetic polymer material with many advantages, has shown great potential in various fields, including agriculture [7,8], clinical medicine [9], drug delivery [10], food industry [11], environmental remediation [12], and cosmetics [13]. Especially, due to its rich carboxyl groups and modifiability, it has shown outstanding results in skin repair and beauty applications to combine γ-PGA with sodium hyaluronate (HA) to prepare injectable hydrogels [14]. Dou et al. introduced a physically crosslinked gelatin network into a covalently crosslinked γ-PGA network. The γ-PGA-GEL double network hydrogel exhibited good compressive strength (38 MPa) and tensile strength (0.27 MPa). In animal experiments, the wound healing rate was 18% higher than that of the control group [15]. Baines et al. demonstrated that γ-PGA can significantly improve the swelling potential of hydrogels. Interestingly, they found that whey protein isolate (WPI) and γ-PGA formed a spherical morphology under some unknown interactions. Therefore, they proposed that if spherical aggregates could continuously induce and establish self-assembly mechanisms, it might be able to further encapsulate drugs [16]. The carboxymethyl chitosan/γ-PGA/tannic acid/carbazole hydrogel, prepared by Mirzamani et al., represents a hydrogel formulation system for the sustained release of carbamazepine. They studied the drug release mechanism through a kinetic model and determined that it is through reducing bacterial colonization in tissues and regulating the inflammatory phase that hydrogels accelerate the healing of MRSA-infected wounds [17].

The wide range of applications for γ-PGA hydrogels has led to the emergence of various preparation methods, such as radiation crosslinking and chemical crosslinking [18,19]. In 1937, Lvanovic first discovered γ-PGA in the capsule of *Bacillus anthracis*. In 1942, Bovarnick extracted γ-PGA from the fermentation broth of *Bacillus subtilis*—the first time γ-PGA was obtained through microbial fermentation. In the 1950s, Record et al. began to study the chemical composition, physical properties, and main functions [20]. Soon after, Hungarian scholars synthesized γ-PGA via peptide synthesis by linking precursor glutamic acid fragments. Researchers have also discovered γ-PGA in Japanese natto, Korean cheonggukjang mucilage, *Bacillus licheniformis*, *Bacillus subtilis* and other sources [21]. Among them, *Bacillus licheniformis* and *Bacillus subtilis* are widely used in related research and industrial fermentation production due to their non-pathogenicity and strong γ-PGA production capacity [22]. There are three methods for γ-PGA production: synthetic methods, enzymatic conversion, and microbial fermentation. The first two have limitations (low yield, impure products, cumbersome operation, and high waste), so microbial fermentation is currently the primary method. However, there are still some issues to be used as a medical aesthetic product or antimicrobial material [16]. Wooyoung Kim et al. used positively charged biomaterials (such as chitosan) that can interact electrostatically with γ-PGA as additives to prepare a γ-PGA-based thermosensitive injectable hydrogel formulation, which changes its state in response to changes in human body temperature. This is undoubtedly an excellent drug delivery method. But micelles formed through non-covalent bonds (such as hydrogen bonds and electrostatic interactions) are likely to be less stable than those formed through chemical cross-linking to form covalent bonds. They may disassemble more quickly in the body, affecting the stability of the material [23]. To address the issues of insufficient crosslinking and stability, some researchers often adopt a dual crosslinking strategy to prepare injectable hydrogels with adjustable crosslinking degrees. However, the introduction of ultraviolet cross-linking may damage bioactive molecules in hydrogels, such as proteins and active peptides, severely affecting their function, and may also cause damage to normal cells and tissues [24,25]. Chemical cross-linking processes may result in residual cross-linking agents, which can cause cytotoxic reactions and greatly limit the promotion and application of materials. In addition, endotoxins are also a safety issue that cannot be ignored in γ-PGA-based materials [26]. Since γ-PGA is a biopolymer produced by microbial fermentation, even low concentrations of endotoxins can trigger severe inflammatory reactions, leading to serious consequences such as fever, shock, and even multiple organ failure. Therefore, endotoxin levels must be strictly controlled. Based on internationally accepted standards, the endotoxin threshold for injectable solutions is typically 0.5 EU/mL [27].

Single γ-PGA micelles serve as promising medical aesthetic materials, though their antimicrobial efficacy remains limited. Increasing their concentration significantly enhances antibacterial activity. Ajayeoba Titilayo A et al. investigated γ-PGA produced by Bacillus species, revealing that composite γPGA polymers exhibit superior bactericidal activity compared to individual γPGA molecules. Moreover, the vast majority of γPGA polymers demonstrated ACE properties [28]. Eman M. Elsayed et al. validated γ-PGA’s inhibitory effects on Gram-positive bacteria (*Staphylococcus aureus* and *Streptococcus pyogenes*) and Gram-negative bacteria (*Klebsiella pneumoniae* and *Escherichia coli*) through in vivo and in vitro experiments. Notably, γ-PGA exhibited stronger inhibitory effects against Gram-positive bacteria [29]. Research indicates that bacterial cell growth can significantly reduce material hydrophilicity and anionic properties [30]. This occurs because carboxyl groups on γ-PGA molecular chains adsorb metal ions like Ca^2+^ and Mg^2+^, impairing bacterial cell membrane stability and enzyme activation, thereby weakening γ-PGA’s antimicrobial efficacy. Given γ-PGA’s water retention and antimicrobial properties, its application in hydrogel dressings is highly suitable, addressing issues such as insufficient moisture retention in wound dressings [31]. Currently, in the antimicrobial application of hydrogel materials, Choi et al. have reported that ultra-high molecular weight γ-PGA hydrogels promote wound healing [32]. Xu et al. also developed electrospun fiber scaffolds based on γ-PGA for preventing hypertrophic scars [33]. Although γ-PGA materials exhibit certain antimicrobial properties, their antibacterial effects are often transient in practical applications due to the absence of antimicrobial drugs [34]. Yang Qu et al. combined γ-PGA with antimicrobial silver nanoparticles, achieving over 90% antibacterial efficacy against *Staphylococcus aureus* and *Escherichia coli*. However, the selection of antimicrobial agents and their loading quantities require further optimization to ensure long-term antibacterial efficacy and biosafety [35].

**Figure 1:**
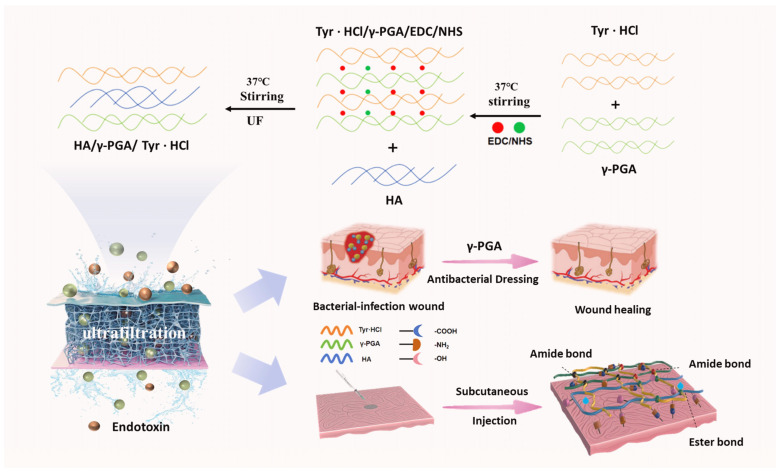
Schematic diagram of γ-PGA-based hydrogel synthesis and applications: First, it is formulated as a medical aesthetic material and injected into the subcutaneous tissue for filling and hydration. Moreover, it is used in combination with antimicrobial agents as an antimicrobial wound dressing.

Therefore, this study developed two γ-PGA-based products (Figure 1): a novel injectable micelle, HA/γ-PGA/tyramine hydrochloride (Tyr·HCl) that can replace hyaluronic acid and a dressing with antibacterial effects. HA/γ-PGA/Tyr·HCl injectable micelles can ensure both stability and safety: Chemical modification of Tyr·HCl to introduce additional cross-linking points increases the cross-linking density of the hydrogel, thereby improving its mechanical stability and durability. HA can regulate material viscosity to suit different needs, and can also adsorb large amounts of water molecules on polysaccharide chains to form a stable hydration layer, thereby increasing the stability and anti-aggregation ability of micelles. HA and γ-PGA, two natural substances, can form stable covalent bonds under the action of EDC/NHS chemical crosslinking agents, and ultimately crosslink with Tyr·HCl to form a triple network structure. These effectively solve the problem of poor micelle stability. At the same time, ultrafiltration redissolution is used to remove endotoxins and residual cross-linking agents, ensuring particle purity and material safety and avoiding cytotoxic reactions. Furthermore, the material can exist in the form of freeze-dried powder after ultrafiltration, which is easier to store [35]. Another product is wound dressing prepared by loading antimicrobial drugs such as penicillin or benzalkonium chloride onto γ-PGA: Its antibacterial efficacy is approximately three times that of existing commercial medical dressings. Therefore, it has a significant competitive advantage in the market.

## 2. Results and Discussion

### 2.1. Injectable Hydrogel

**Figure 2:**
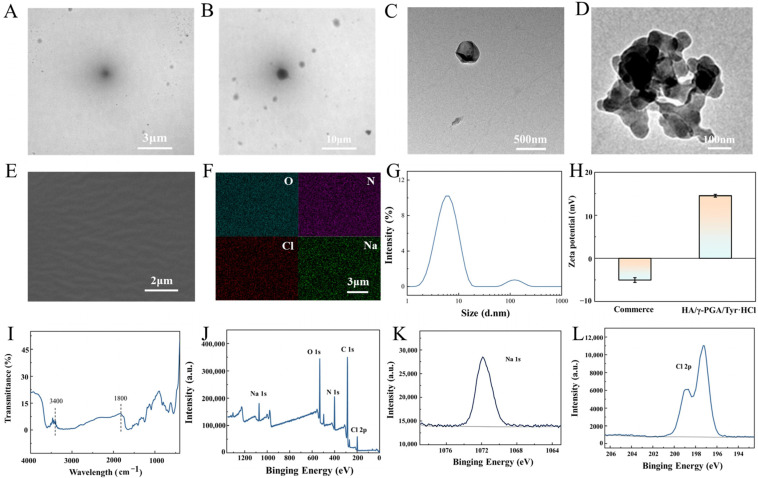
(**A**) TEM image of HA/γ-PGA/Tyr·HCl after ultrafiltration; (**B**) TEM image of HA/γ-PGA/Tyr·HCl before ultrafiltration; (**C**) TEM image of a single particle of HA/γ-PGA/Tyr·HCl after ultrafiltration; (**D**) Enlarged TEM image of a single particle; (**E**) SEM image of γ-PGA hydrogel precursor; (**F**) Distribution of O, N, Cl, and Na Elements in γ-PGA hydrogel precursor; (**G**) DLS image of γ-PGA hydrogel; (**H**) Zeta plots of commercial hydrogel and γ-PGA hydrogel; (**I**) FTIR spectrum of γ-PGA hydrogel; (**J**–**L**) XPS spectra of γ-PGA hydrogel.

The polyglutamic acid is provided as raw material and subsequently polymerized/cross-linked by Jilin Folialux Bio-tech Co., Ltd. [36], and synthesized into injectable micelles according to the test method. To investigate the changes in γ-PGA during micelle synthesis, it is necessary to compare the morphologies of the commonly available sodium hyaluronate composite solution for injection on the market (Haiti brand) and HA/γ-PGA/Tyr·HCl: From the TEM image of commercial products, it can be seen that the particles are spherical, with a particle size of approximately 100 nm. The TEM image of the synthesized γ-PGA hydrogel also showed similar results, with a particle size of approximately 100 nm, forming a stable spherical micelle structure. Figure 2A and Figure 2B show TEM images of HA/γ-PGA/Tyr·HCl after ultrafiltration and without ultrafiltration, respectively. It can be seen that the ultrafiltration process did not significantly affect the structure of the micelles themselves. The particle size of individual gel particles in the triple cross-linked structure hydrogel increased to approximately 400 nm (Figure 2C), which provide greater stability and viscosity. The internal structure of the triple-crosslinked hydrogel particles is similar to a chain-like agglomeration structure (Figure 2D), which makes HA/γ-PGA/Tyr·HCl more stable. This result demonstrates the good molding properties of hydrogels. Figure 2E shows the SEM image of the γ-PGA hydrogel precursor solution, revealing a highly uniform hydrogel. Figure 2F and Figure S6 show the elemental distribution of micelle samples before and after ultrafiltration. The types of elements in the micelle solution did not change before and after ultrafiltration, and the micelle particles remained in the redissolved solution after ultrafiltration. At the same time, observation of the dense freeze-dried powder blocks formed by micelle ultrafiltration also demonstrates their freeze-drying stability and structural retention capabilities.

Compared to commercial hyaluronic acid and γ-PGA micelle particles already published, HA/γ-PGA/Tyr·HCl have smaller particle sizes and better dispersibility (Figure 2G). So they exhibit better biocompatibility and tissue penetration capability. This not only effectively reduces immune responses but also enhances drug delivery efficiency. The γ-PGA hydrogel particles also exhibit a higher zeta potential (Figure 2H). This is mainly because chemical modification (NHS/EDC cross-linking) introduces positive charges, making it difficult for them to aggregate in the dispersion system, resulting in better stability of the entire system. In Fourier transform infrared spectroscopy (FTIR) analysis (Figure 2I), the appearance of the H-O stretching vibration peak at 3400 cm^−1^ indicates that the hydroxyl group is bound to water molecules, while the peak at 1800 cm^−1^ is caused by the carboxyl group binding to Na^+^ to form a carboxylate, which in turn leads to the C=O stretching vibration peak. Additionally, X-ray photoelectron spectroscopy (XPS) analysis was performed on HA/γ-PGA/Tyr·HCl (Figure 2J–L). In this analysis, Na was selected to represent HA and Cl was selected to represent Tyr·HCl. It can be clearly seen that a binding energy peak has formed between Cl and Na. This proves that Tyr·HCl and HA have been successfully combined into the material system.

**Figure 3:**
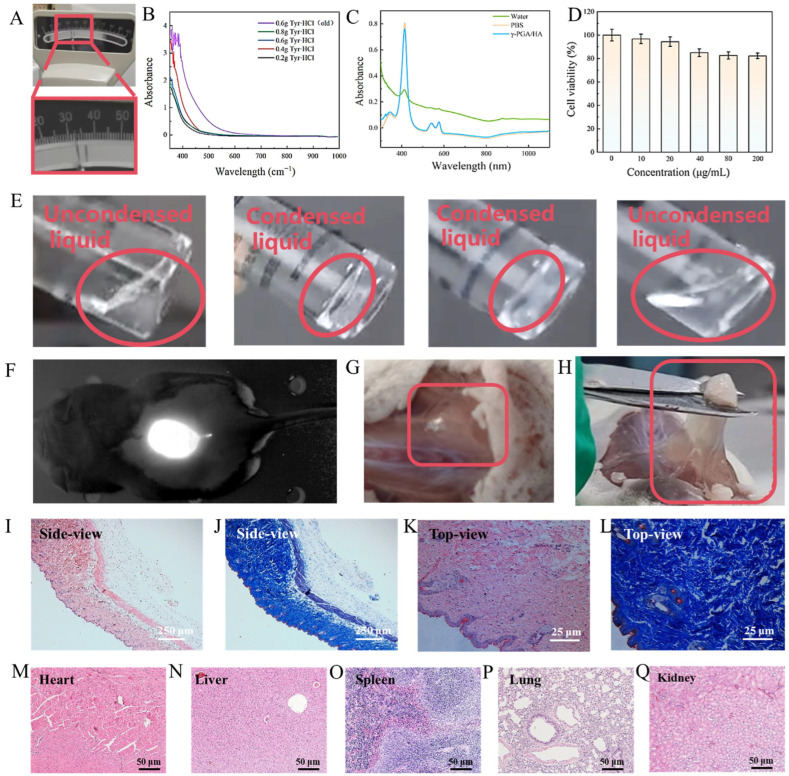
(**A**) Viscosity of HA/γ-PGA/Tyr·HCl precursor solution; (**B**) UV absorption spectra of γ-PGA hydrogels with different Tyr·HCl concentrations, and the UV absorption spectrum of the hydrogel containing 0.6 g Tyr·HCl after standing for a period of time; (**C**) Absorption spectra of hemolysis rate of γ-PGA hydrogel; (**D**) CCK8 cytotoxicity of γ-PGA micelles; (**E**) Photographs showing interference of micellar material endotoxin samples and gel-based test results. From left to right, they specifically represent: represents the endotoxin-free sample (blank control); represents the positive control group with endotoxin at 2λ; represents the positive control group with endotoxin at 2λ; represents the endotoxin-free negative control group; (**F**) Subcutaneous filling imaging of mouse with γ-PGA micelles; (**G**) Subcutaneous injection filling effect of γ-PGA micelles; (**H**) Elasticity effect of γ-PGA micelles; (**I**,**J**) Side-view images of skin H&E and Marson after subcutaneous injection of γ-PGA micelles; (**K**,**L**) Top-view images of skin H&E and Marson after subcutaneous injection of γ-PGA micelles; (**M**–**Q**) H&E of major organs (heart, liver, spleen, lung, and kidney) after subcutaneous injection of γ-PGA micelles.

We also tested the relevant properties of the HA/γ-PGA/Tyr·HCl precursor solution. In industrial production, similar hydrogel precursor solutions typically exhibit viscosities of 30–40 mPa·s. Subsequent addition of HA adjusts hydrogel viscosity to suit practical applications. Therefore, we measured the viscosity of the HA/γ-PGA/Tyr·HCl precursor solution. Under room temperature (approximately 20 °C) conditions, its viscosity was 32 mPa·s (Figure 3A), consistent with previous studies. Figure 3B shows the effect of different Try·HCl addition amounts on the material. It can be seen that the results of each group do not differ greatly. In addition, drying treatment can reveal the film-forming properties of the material. Compared with commercial hyaluronic acid, γ-PGA micelles have better film-forming properties (Figure S7) and can provide more stable support and protection.

HA/γ-PGA/Tyr·HCl, as a medical aesthetic material, must meet the requirement of a hemolysis rate below 5%. Figure 3C shows the absorbance values at 545 nm for the experimental group, positive control (blood + water), and negative control (blood + PBS). Calculations reveal that the hemolysis rate of HA/γ-PGA/Tyr·HCl is 3.25%, far below the 5% threshold. This indicates that the material exhibits excellent blood compatibility. The results of the cytotoxicity test are shown in Figure 3D. The survival rates of fibroblasts in different concentration groups were all above 80%. Among them, the survival rate of cells at 125 μg/mL reaching as high as 95%. This indicates that the material has low cytotoxicity and has little effect on cell survival and proliferation. In addition, medical aesthetic materials that come into direct contact with blood or tissue must have endotoxin levels strictly controlled within safe limits. Therefore, the presence of endotoxin was detected by using Limulus reagent to produce an agglutination reaction with endotoxin. Following the operations under the Limulus reagent sensitivity verification test, it can be clearly observed that groups d and a did not agglutinate (negative), while groups c and b agglutinated (positive) (Figure 3E). At this point, it can be concluded that the endotoxin content is below 0.5 EU/mL, indicating that HA/γ-PGA/Tyr·HCl has a high level of safety. Some existing studies have made similar statements, indicating that endotoxin levels below 0.5 EU/mL can demonstrate the safety of the material.

After confirming the physical and chemical properties and safety of HA/γ-PGA/Tyr·HCl micelles, this study needed to evaluate their injection effects through animal experiments. All animal experiments were conducted in accordance with the guidelines of the Institutional Animal Care and Use Committee, and subcutaneous injections and biopsy tests were performed in accordance with the authorization agreement of Jilin University (JLUKQ #SY202105013). To get a clearer picture of how the micelles form in mice, we mixed them with the fluorescent dye ICG and injected them under the skin of mice, achieving small animal imaging (Figure 3F). The injection results (Figure 3G) showed that the micelles were biocompatible and filled the subcutaneous space well: It forms a hydrogel layer beneath the skin, producing a filling effect. Subsequently, cutting this gel (Figure 3H) further demonstrates excellent elasticity and moldability. These comprehensive results indicate that it can form a stable filling effect subcutaneously. H&E staining and Masson images of the subcutaneous skin (Figure 3I–L) show normal tissue structure and collagen fiber arrangement. We can also observe a reduction in inflammatory cells and the formation of new tissue, which further confirms the safety and efficacy of the material. Figure 3M–Q show H&E of mouse internal organs (heart, liver, spleen, lungs, and kidneys) after injection of the micelles. As can be seen, the H&E did not show any abnormal tissue reactions, indicating that the material has good biocompatibility.

### 2.2. Dressings

**Figure 4:**
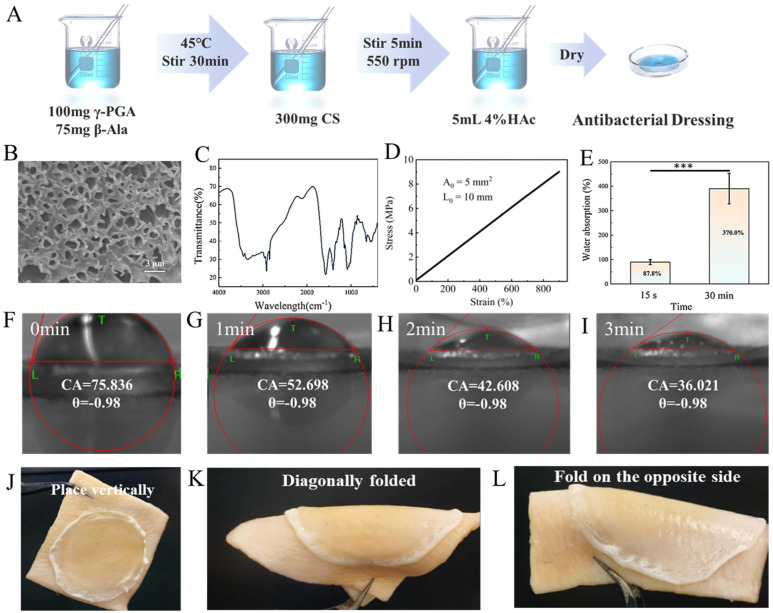
(**A**) Schematic diagram of the synthesis process of γ-PGA hydrogel dressings; (**B**) SEM image of γ-PGA hydrogel dressings; (**C**) Infrared spectrum of the γ-PGA hydrogel dressing precursor solution; (**D**) Tensile properties of γ-PGA hydrogel dressings; (**E**) Water absorption of γ-PGA hydrogel dressings (*** *p* < 0.001) (*n* = 4); (**F**–**I**) Effect of water contact angle changes in γ-PGA hydrogel dressings; (**J**–**L**) Adhesion test results of γ-PGA hydrogel dressings.

Preparing γ-PGA hydrogels (Figure 4A) and loading them with benzalkonium chloride (BAC) yields antimicrobial dressings. As shown in Figure 4B, the γ-PGA hydrogel dressing exhibits a porous network structure with uniformly sized and widely distributed pores. The pore structure provides ample space for water absorption and retention, allowing gases such as oxygen and carbon dioxide to pass through. This helps maintain the wound microenvironment, promote cellular metabolism, and facilitate tissue regeneration, thereby accelerating the wound healing process. Figure 4C shows the FTIR spectrum of the dressing, clearly revealing characteristic vibration peaks at 3415 cm^−1^ and 1620 cm^−1^. This can be attributed to interactions between electrostatic polymerization and hydrogen bonding. Specifically, the generation of electrostatic interactions is directly related to the introduction of β-alanine (β-Ala), while hydrogen bonding interactions are due to the presence of polar groups such as carboxyl, amino, and hydroxyl groups in the chitosan (CS) and γ-PGA hydrogel system. The presence of these characteristic peaks fully confirms the successful synthesis of the dressing.

As an antimicrobial dressing, the tensile and adhesive properties of γ-PGA hydrogel dressings are of great importance. Tensile tests (Shanghai Hualong Testing Instruments Co., Ltd. (Shanghai, China)) were conducted on γ-PGA hydrogel dressings using a universal testing machine, with results shown in Figure 4D. The original dimensions of the hydrogel are 10 mm in length, 10 mm in width, and approximately 0.5 mm in thickness. And the results indicated that the dressing remains in the elastic stage when stretched from 10 mm to 80 mm. Its elongation at break reached approximately 850%. Compared to existing hydrogel research, the elongation at break of the Au@Ag/PC hydrogel dressing is only 715% [37]. This indicates that the hydrogel dressing possesses excellent elasticity and extensibility, capable of maintaining its integrity even after undergoing significant stretching without breaking. Additionally, the γ-PGA hydrogel dressing exhibits outstanding water absorption performance (Figure 4E and Figure S14). When immersed in deionized water, the water absorption rate reached 87.8% after 15 s and rapidly increased to 370% after 30 min. The water absorption of γ-PGA hydrogel dressings primarily stems from their internal polymer network structure. Additionally, γ-PGA is rich in carboxyl (-COOH) groups, which form hydrogen bonds in water. These enable the material to rapidly absorb large amounts of water in a short time and maintain stable water absorption capacity over an extended period. The water contact angle can reflect the hydrophilic properties of a material. As shown in Figure 4F–I, the water contact angle of the γ-PGA hydrogel dressing decreased significantly over time, dropping from 75.836° to 36.021° in just 3 min. This trend indicates that the surface of the hydrogel dressing has excellent hydrophilicity, enabling it to rapidly reduce the water contact angle and thereby enhance wettability. To assess the material’s adhesion, we applied it to animal tissue (such as pig skin) and conducted adhesion tests under conditions simulating tissue fluid with a single drop of water. The experimental results (Figure 4J–L) show that the hydrogel dressing does not detach even when the pig skin is deformed. This indicates that it possesses excellent adhesion properties.

**Figure 5:**
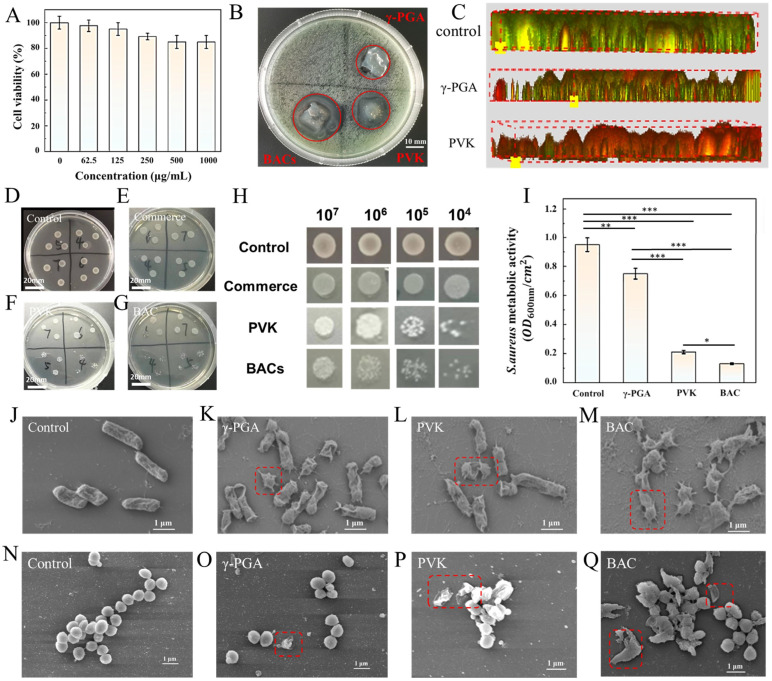
(**A**) Cytotoxicity of the hydrogel dressing precursor solution; (**B**) Antimicrobial zone test results after processing with different materials (Control group; γ-PGA; γ-PGA loaded with BACs; γ-PGA loaded with PVK); (**C**) Bacterial viability staining test results after processing with different materials (Control group; γ-PGA; γ-PGA loaded with PVK); (**D**–**G**) Antimicrobial efficacy after processing with different materials in CFU experiments (4, 5, 6, 7: The initial bacterial density is 10^4^, 10^5^, 10^6^, 10^7^ CFU, respectively); (**H**) Magnified view of CFU experimental colonies for each group; (**I**) Results of MTT bacterial experiments for each group (*: *p* < 0.05; **: *p* < 0.01; ***: *p* < 0.001) (*n* = 3); (**J**–**M**) SEM experimental results for *E.coli* after processing with different materials; (**N**–**Q**) SEM experimental results for *S. aureus* after processing with different materials.

To ensure its safety, the cytotoxicity of the γ-PGA hydrogel dressing also needs to be tested. For convenience, the precursor solution of the dressing was combined with antimicrobial drugs for testing. The results are shown in Figure 5A. When the material concentration was below 1000 μg/mL, the cell viability of all groups is above 80%. This indicates that the material has low cytotoxicity and does not affect normal cells.

To better evaluate the antimicrobial efficacy of the dressings, this experiment was divided into four groups: commercial hydrogel dressing (Control), γ-polyglutamic acid hydrogel dressing (γ-PGA), hydrogel dressing combined with penicillin (PVK), and hydrogel dressing combined with benzalkonium chloride (BACs). Each group evaluated its inhibitory effect on bacteria through a zone of inhibition test. As can be seen in Figure 5B, the γ-PGA group had a small and inconspicuous zone of inhibition (18.57 ± 0.39 mm), while the PVK and BAC groups had obvious zones of inhibition, with the BACs group having a larger zone of inhibition. The BACs group and PVK group exhibited inhibition zone diameters of 25.71 ± 0.73 mm and 20.71 ± 0.89 mm, respectively. In existing studies, the antibacterial zone concentrations of these materials were only 15.96 ± 1.36 mm and 15.70 ± 0.95 mm. This undoubtedly demonstrates that γ-PGA hydrogel dressings loaded with BACs and PVK exhibit superior antimicrobial properties. Figure 5C shows the results of the bacterial viability staining experiment. The bacterial layers in the control group, γ-PGA group, and PVK group became increasingly thinner as the experiment progressed, and a large number of dead bacteria (red) were observed in the PVK group. In the subsequent CFU (colony-forming unit) bacterial experiment, similar results were obtained (Figure 5D–G). And magnified views for each group are shown in Figure 5H: the bacterial density in the control group was extremely high, with limited antibacterial efficacy. The bacterial density in the two drug combination groups was significantly reduced, indicating that the combination of γ-PGA with antibacterial drugs can significantly enhance the antibacterial efficacy of the dressing. In addition, each group can also conduct MTT bacterial experiments to quantitatively analyze their inhibitory effects on *S. aureus*. The optical density (OD) values at 600 nm for each group are shown in Figure 5I. It can be seen that: The commercial hydrogel dressing in the control group has almost no antibacterial effect; γ-PGA has a weak antibacterial effect, but its antibacterial performance is significantly enhanced when combined with antibacterial drugs. γ-PGA loaded with PVK reduced the cellular activity of *S*. *aureus* by 84.2%. These results indicate that hydrogel dressings have some antibacterial activity on their own, but their antibacterial effect is significantly enhanced when combined with antibacterial drugs (especially penicillin). In existing experiments, similar results have been demonstrated. The γ-PGA hydrogel dressing exhibits antibacterial efficacy exceeding 80% against *S*. *aureus*, proving its excellent antimicrobial performance. In addition, the antibacterial effect of the material can be evaluated using a zone of inhibition test. Observing *E.coli* and *S. aureus* under a scanning electron microscope allows for direct visualization of changes in bacterial morphology (Figure 5J–Q). As expected, the morphology of bacteria in the two drug combination groups was significantly disrupted, indicating that the antibacterial drugs interfered with the normal physiological functions of the bacteria, leading to damage to the bacterial cell structure or even death.

## 3. Materials and Methods

### 3.1. Materials

The polyglutamic acid is provided as raw material and subsequently polymer-ized/cross-linked by Jilin Folialux Bio-tech Company Limited (Changchun, China) [36]. Tyramine hydrochloride was purchased from Shanghai McLean Biochemistry and Technology Company Limited (Shanghai, China). Ethyldimethylaminopropyl Carbodiimide was purchased from Shanghai Aladdin Biochemical Science and Technology Company Limite (Shanghai, China). N-Hydroxysuccinimide was purchased from Shanghai McLean Biochemistry and Technology Company Limited (Shanghai, China). Sodium hyaluronate was purchased from Huaxi Biotech Company Limited (Jinan, China), specifically classified as medical device hyaluronic acid sodium-HA-EP2. Amicon^®^ Ultra-15 ultrafiltration centrifuge tubes were purchased from Millipore Company Limited (Hangzhou, China). H&E staining and Masson’s trichrome staining reagents were purchased from Beijing Solarbio Biotechnology Company Limited (Beijing, China).

### 3.2. Preparation of Injectable Micelles

At room temperature, 3–9 parts by weight of γ-polyglutamic acid and 2–7 parts by weight of tyramine hydrochloride were dissolved in 40–60 parts by weight of distilled water. After they were completely dissolved, 1–5 parts by weight of 1-(3-dimethylaminopropyl)-3-ethylcarbodiimide hydrochloride (EDC) was added. The mixture was stirred thoroughly to form the first solution. Next, an equal amount of N-hydroxysuccinimide (NHS) was added to the first solution (Figure S1A). This step helped obtain the second solution. Subsequently, 0.1–0.5 parts by weight of a viscosity modifier (sodium hyaluronate) was incorporated into the second solution. The mixture was stirred at 37 °C. A magnetic stirrer or water bath was used for stirring. The stirring continued until the modifier was completely dissolved and uniformly dispersed. The resulting solution formed an improved hydrogel with a triple-crosslinked structure (Figure S1B). Thereafter, the triple-crosslinked hydrogel solution was transferred to an ultrafiltration centrifuge tube (Figure S2). It was then subjected to ultrafiltration and centrifugation. Finally, the ultrafiltered solute was redissolved in water. This formed an injectable γ-polyglutamic acid-based hydrocapsule (Figure S1C).

### 3.3. Preparation of Freeze-Dried Powder

Impurities were removed from the solution through filtration or centrifugation, a process intended to purify the system. Next, the solution concentration underwent adjustment via evaporation or dilution, a step designed to facilitate subsequent freeze-drying. A shaping agent was incorporated, with the mixture thoroughly stirred before transfer to appropriate containers. The solution underwent rapid freezing at low temperatures, a treatment aimed at promoting the formation of small ice crystals—initial freezing occurred at −80 °C overnight. Test tube caps were opened and covered with hole-punched aluminum foil prior to being returned to the freezer. Samples were prepared in a freeze-dryer, with the resulting powder collected in a sterile, dry environment to prevent contamination or moisture absorption. Finally, the powder was sealed in moisture-proof, light-resistant packaging, which was then stored in a cool, dry environment; temperature and humidity fluctuations were avoided to maintain product stability.

### 3.4. Preparation of Antimicrobial Dressings

Next, 100 mg of γ-PGA and an appropriate amount of alanine were dissolved in 15 mL of 1 × 10^−5^ mol/L NaOH solution (pH 9.0), with stirring at 45 °C and 550 rpm for 30 min to ensure full dissolution. Then, 300 mg of chitosan (CS) was then added to the solution, followed by stirring at 550 rpm for 5 min—a step to achieve uniform distribution. Next, 5 mL of 4% glacial acetic acid solution was added dropwise at 10 drops per minute to the CS/γ-PGA mixture, with continuous stirring at 550 rpm and 45 °C for 45 min to promote reaction. The precursor slurry, ready for shaping, was slowly poured into a 9 cm diameter Petri dish and heat-dried overnight at 45 °C, a process forming the precursor hydrogel. Immersion in 15 mL of 0.1 mol/L NaOH solution for 2 h was followed by rinsing with deionized water until the eluate’s pH remained constant, a treatment to neutralize excess acid. Finally, the hydrogel was placed at 45 °C until constant weight, completing the preparation of the hydrogel material.

A 1% benzalkonium chloride (BAC) solution was prepared first. The hydrogel dressing was immersed in this solution, with BAC loading controlled to 1–3% of the hydrogel’s weight. Soaking proceeded at room temperature for 2 h, a duration ensuring uniform distribution of BAC throughout the hydrogel. Post-soaking, the hydrogel surface was gently rinsed with deionized water to remove any unadsorbed BAC. Finally, the hydrogel was left to air dry at room temperature.

### 3.5. Test of Viscosity

The viscosity of HA/γ-PGA/Tyr·HCl precursor solution was tested using NDJ-79 Rotational Viscometer (Shanghai Genggeng Instrument Equipment Co., Ltd. (Shanghai, China)). The test liquid (the second solution) was slowly poured into Test Vessel II Until the liquid covered the edge of the plate. Rotor II corresponds to the flat plate measurement system, with a diameter of approximately 24 mm. The rotational speed is set to 7.5 rpm, corresponding to a shear rate of approximately 1.5–15 s^−1^ (refer to the rotor constant table for specifics). With the rotor fully immersed in the liquid, the test vessel was placed on the instrument’s support bracket, while the rotor was simultaneously suspended on the instrument’s coupling—ensuring complete submersion of the rotor at this stage. The motor was then started. Rotor rotation, which may be accompanied by wobbling, required adjustment: the test container was moved back and forth or side to side on the bracket to achieve concentric alignment with the rotor, a maneuver stabilizing the pointer for accurate reading.

### 3.6. Test of Cytotoxicity

L929 cells were cultivated in medium containing 10% serum until reaching 80–90% confluence. Trypsin was used for cell detachment, after which the cell suspension was diluted and 100 μL aliquots were added to each well of a 96-well plate. The plate was incubated at 37 °C in a cell culture incubator for 24 h, a period allowing for cell attachment. Predetermined drug concentrations were then added to each well, with incubation continued at 37 °C for another 24 h. Post-treatment, 10 μL of CCK-8 reagent was added to each well, followed by incubation at 37 °C for an additional 12 h. Finally, absorbance at 450 nm was measured using a microplate reader, with subsequent data analysis and calculation.

### 3.7. Test of Hemolysis Rate

First, blood samples were collected by selecting suitable animals (such as mice) as blood donors and obtaining fresh blood. A red blood cell suspension was prepared through centrifugation and washing of the blood, with centrifugation and washing repeated to ensure thorough cleaning.

Next, the experimental setup was prepared, including a positive control group, a negative control group, and experimental groups. In the experimental groups, micelle material was brought into direct contact with the red blood cell suspension at predetermined concentration and volume ratios. The test material, once mixed with the red blood cell suspension, was incubated at 37 °C for a specified duration. Following incubation, hemolysis was observed via centrifugation. Qualitative and quantitative analysis was conducted by measuring absorbance at a specific wavelength (e.g., 545 nm) using a spectrophotometer, with absorbance (OD value) determined in the supernatant and calculated using the following formula:

$$Hemolysis\;Rate\left(\%\right)=\frac{{OD}_{Sample}-{OD}_{Control(-)}}{{OD}_{Control(+)}-{OD}_{Control(-)}}\times 100\%$$

### 3.8. Test of Endotoxin

Given the large molecular weight of endotoxins, an ultrafiltration tube with a 100,000 molecular weight cutoff was selected. The prepared HA-γ-PGA micelle pre-solution was added to the ultrafiltration centrifuge tube, which—after capping—was placed into the centrifuge rotor and balanced. Centrifugation was conducted using a swing-bucket rotor at an appropriate centrifugal force for 15 to 60 min. For recovery of the concentrated product, a pipette (tip) was inserted into the inner tube of the ultrafiltration centrifuge tube, with the tip moved back and forth to aspirate the sample, a technique ensuring complete recovery. The filtrate remained storable in the centrifuge outer tube.

### 3.9. Test of Endotoxin Interference

Solutions A, B, C, and D were prepared according to Table 1, with the test solution selected as one free of detectable endotoxins and not exceeding the maximum valid dilution (MVD).

Procedures outlined under the Limulus Reagent Sensitivity Verification Test were as follows:

The test is valid only when all parallel tubes of solution A and negative control solution D yield negative results, and results for series solution C fall within the sensitivity verification range of the horseshoe crab reagent. The geometric mean of the endpoint concentrations for series solutions C and B (Es and Et) was calculated as follows:

$$Es=antilg\frac{\sum Xs}{4}$$

$$Et=antilg\frac{\sum Xt}{4}$$

In the equation, Es and Et denote the logarithmic endpoint concentrations (lg) of Series Solution C and Solution B, respectively. The test substance exhibits no interference at the concentration in question when Es falls within the range of 0.5λ–2λ (inclusive) and Et lies within 0.5 Es–2 Es (inclusive). Should interference occur at a dilution factor below the MVD, the test substance solution is further diluted to a factor not exceeding the MVD, with the interference test repeated thereafter.

### 3.10. Test of Gel Limit

Solutions A, B, C, and D were prepared according to Table S1, with solutions A and B formulated using the test solution at the MVD factor—confirmed to be free of interference. Procedures outlined under the Limulus Reagent Sensitivity Verification Test were followed.

Result Interpretation: Observations were made after 60 ± 2 min of incubation. (1) The test is valid if both parallel tubes of negative control solution D show negative results, both parallel tubes of positive control solution B yield positive results, and both parallel tubes of positive control solution C return positive results. (2) The test sample is deemed compliant when both parallel tubes of Solution A are negative; it is deemed noncompliant if both parallel tubes of Solution A are positive. In cases where one parallel tube of Solution A is positive and the other is negative, a retest is required. For the retest, four parallel tubes of Solution A are prepared: the test sample is considered compliant if all parallel tubes are negative, otherwise noncompliant.

### 3.11. Test of Subcutaneous Injection in Mice

12 Kunming mice were prepared and acclimated pre-experiment to ensure good health. The 12 mice were divided into an experimental group and a control group, with 6 mice in each group. The experiment was repeated three times to ensure statistical significance. All animal procedures were performed under inhalation anesthesia. The experiment was approved by the Animal Ethics Committee of Jilin University and strictly conducted in accordance with national regulations for laboratory animal management.

The prepared micelles were diluted to the appropriate concentration according to the predetermined dosage. In a biosafety cabinet, the micelle solution was drawn up with a sterile syringe; after restraining the mouse, subcutaneous injection was administered into its back at a steady rate, a technique to avoid micelle rupture. Post-injection, the mice were observed, with any abnormal reactions recorded. Regular monitoring of the mice was conducted to track behavioral changes, weight fluctuations, and physiological responses. For assessment of subcutaneous effects, selected mice underwent necropsy to observe in vivo micelle distribution.

Histological study methods: Skin from the injection site and major organs (heart, liver, spleen, lungs, kidneys) were collected, fixed in 4% paraformaldehyde for 24 h, embedded in paraffin, sectioned (4 μm thick), and subjected to hematoxylin and eosin (H&E) staining and Masson’s trichrome staining. Staining reagents were purchased from Beijing Solarbio Science & Technology Co., Ltd. (Beijing, China), and staining procedures were performed according to the manufacturer’s instructions.

### 3.12. Statistical Analysis

Microsoft Excel 2021 was used for data collection. Origin 2024 was used for the analysis of material properties. Microsoft PPT 2021 was used for graphical representation. SPSS 26.0 software was used to perform statistical analysis on all data. ImageJ1.54f was used for image analysis, and Microsoft PPT 2021 was used for image assembly. All data are presented as mean ± standard deviation (SD). Statistical significance was determined using the *t*-test and one-way ANOVA. Between-group variance was tested for significance, with statistical thresholds set at *p* < 0.05, *p* < 0.01 and *p* < 0.001.

## 4. Conclusions

In summary, this study addresses the critical challenge of balancing micelle stability against endotoxin control in hydrogel design. We developed a novel composite micelle system based on γ-PGA, HA, and Tyr·HCl, which significantly enhances structural stability through a triple-network architecture formed via EDC/NHS crosslinking. In this system, HA provides strong hydrophilicity and forms a hydration layer that prevents aggregation. Tyr·HCl introduces additional cross-linking sites to strengthen the network. Furthermore, ultrafiltration and redissolution processes effectively remove impurities and reduce endotoxin levels, ensuring high material purity and biosafety. On this basis, we further fabricated antibacterial dressings by loading penicillin or benzalkonium chloride into the γ-PGA hydrogel, which demonstrated superior antibacterial performance compared to existing medical dressings. This design not only fulfills the demands of industrial production with cost efficiency, but also enhances the commercial potential of γ-PGA. Our findings offer innovative solutions for applying γ-PGA-based hydrogels in medical aesthetics and wound management and provide a valuable reference for the development of γ-PGA as a drug delivery platform in clinical medicine.

## Figures and Tables

**Table 1:** Preparation of Solutions for the Gel-Based Interference Test.

	Endotoxin Concentration/ Solution Containing Added Endotoxin	Dilution Solution	Dilution Ratio	Concentration of Endotoxins Contained	Parallel Experiments
A	None/Test solution				
B	2λ/Test solution	Test solution	1	2λ	4
			2	1λ	4
			4	0.5λ	4
			8	0.25λ	4
C	2λ/Inspection water	Inspection water	1	2λ	4
			2	1λ	4
			4	0.5λ	4
			8	0.25λ	4
D	None/Inspection water	—	—	—	2

Note: A denotes the test solution; B represents the interference test series; C indicates the sensitivity verification series for the horseshoe crab reagent; D signifies the negative control.

## Data Availability

The data that support the findings of this study are available on request from the corresponding author.

## Supplementary Materials

The following supporting information can be downloaded at: https://www.mdpi.com/article/10.3390/molecules30214205/s1. Figure S1: γ-PGA hydrogels at different stages; Figure S2: Actual image of ultrafiltration tube; Figure S3: Actual Photo of Freeze-Dried Powder Effect; Figure S4: Actual image of Hi-Body brand hyaluronic acid nano-complex solution; Figure S5: SEM images of different materials; Figure S6: SEM (A) and mapping (B) of γ-PGA hydrogel precursor after ultrafiltration; Figure S7: Film-forming effect of γ-PGA micelles (A) and Hi-Body brand sodium hyaluronate composite solution (B); Figure S8: DLS image of commercial hyaluronic acid; Figure S9: Endotoxin Detection Reagents and Methods; Figure S10: Centrifuged Supernatant Physical Image; Figure S11: H&E staining of subcutaneously injected commercial injectable micelles in animal visceral tissue sections; Figure S12: Actual image of a commercial hydrogel dressing; Figure S13: Photograph of CS/γ-PGA/β-Ala hydrogel (A) and schematic diagram of manual stretching (B); Figure S14: Actual image (A) and data chart (B) of water absorption capacity for γ-PGA hydrogel dressing (*p* < 0.001) (*n* = 4); Table S1: Preparation of Solutions for the Gel Limit Test.
