# Supplementary material for: A Safe and Multifunctional γ-PGA Hydrogel Platform: Endotoxin-Controlled Injectable Fillers and Antimicrobial Wound Dressings
Source: Molecules. 2025 Oct 28;30(21):4205. doi: 10.3390/molecules30214205 (PMC12610730; doi:10.3390/molecules30214205)
Supplement: Supplementary file 1 [file molecules-30-04205-s001.zip › molecules-3884950-supplementary.pdf]

Supplementary Table

Table S1. Preparation of solutions for the gel limit test.

	Endotoxin concentration/<br>Preparation of endotoxin solution	Parallel<br>experiments
A	None/Test solution	2
B	2 λ /Test solution	2
C	2 λ /Inspection water	2
D	None/Inspection water	2

Note: A denotes the test sample solution; B denotes the positive control for the test sample; C denotes the positive control; D denotes the negative control.

Supplementary Figures

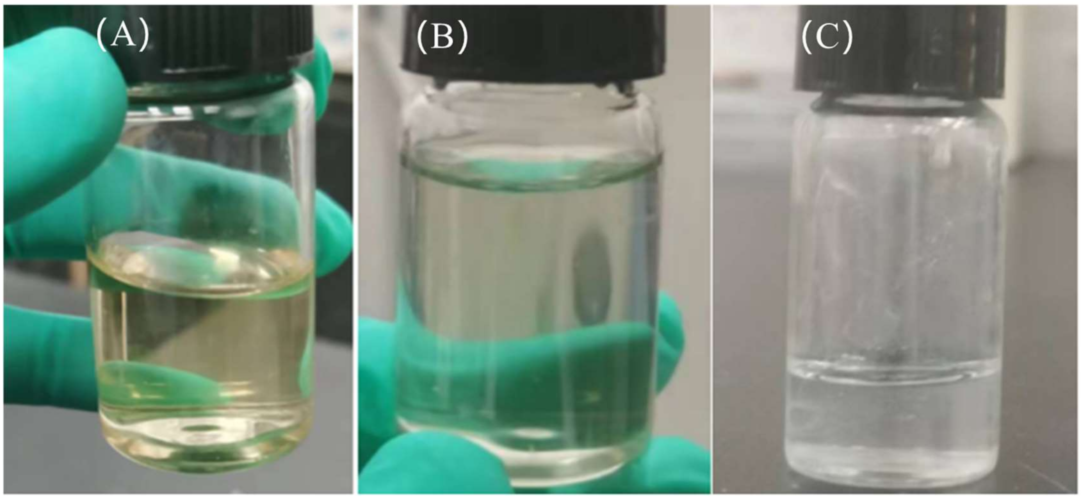

Figure S1. (A) Precursor solution of γ-PGA and crosslinking agent; (B) hydrogel solution with triple crosslinking structure; (C) γ-PGA hydrogel obtained after ultrafiltration and redissolution.

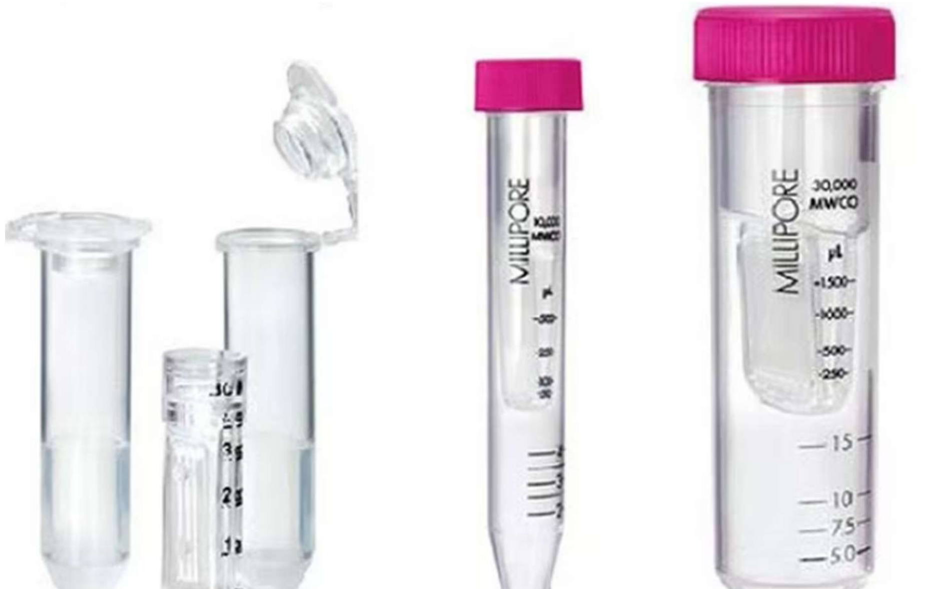

Figure S2. Actual image of ultrafiltration tube.

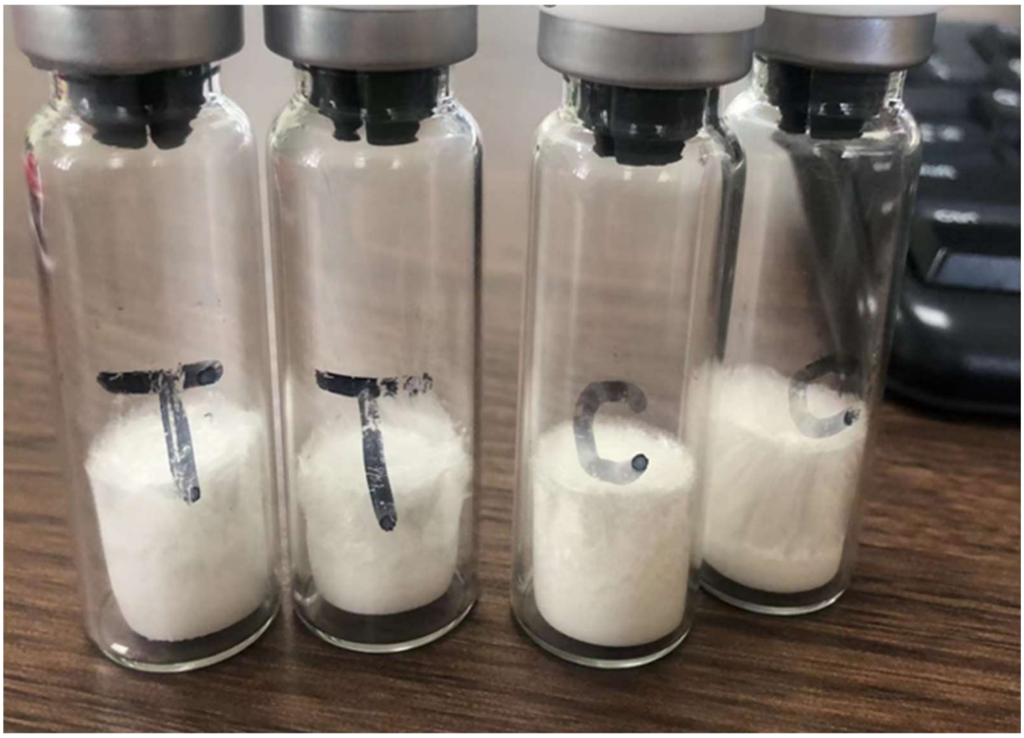

Figure S3. Actual photo of freeze-dried powder effect.

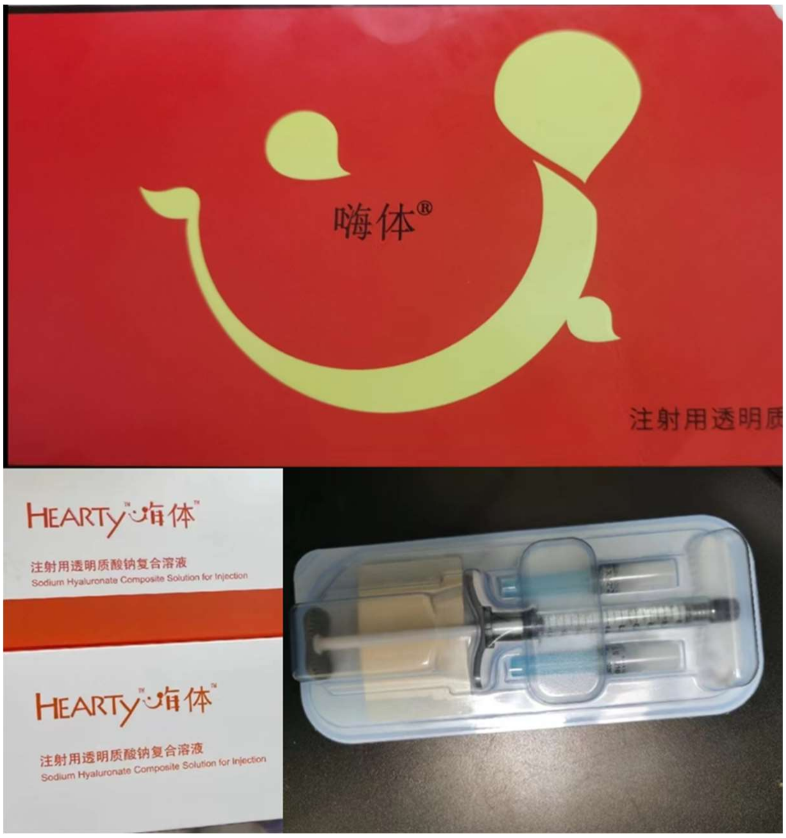

Figure S4. Actual image of Hi-Body brand hyaluronic acid nano-complex solution.

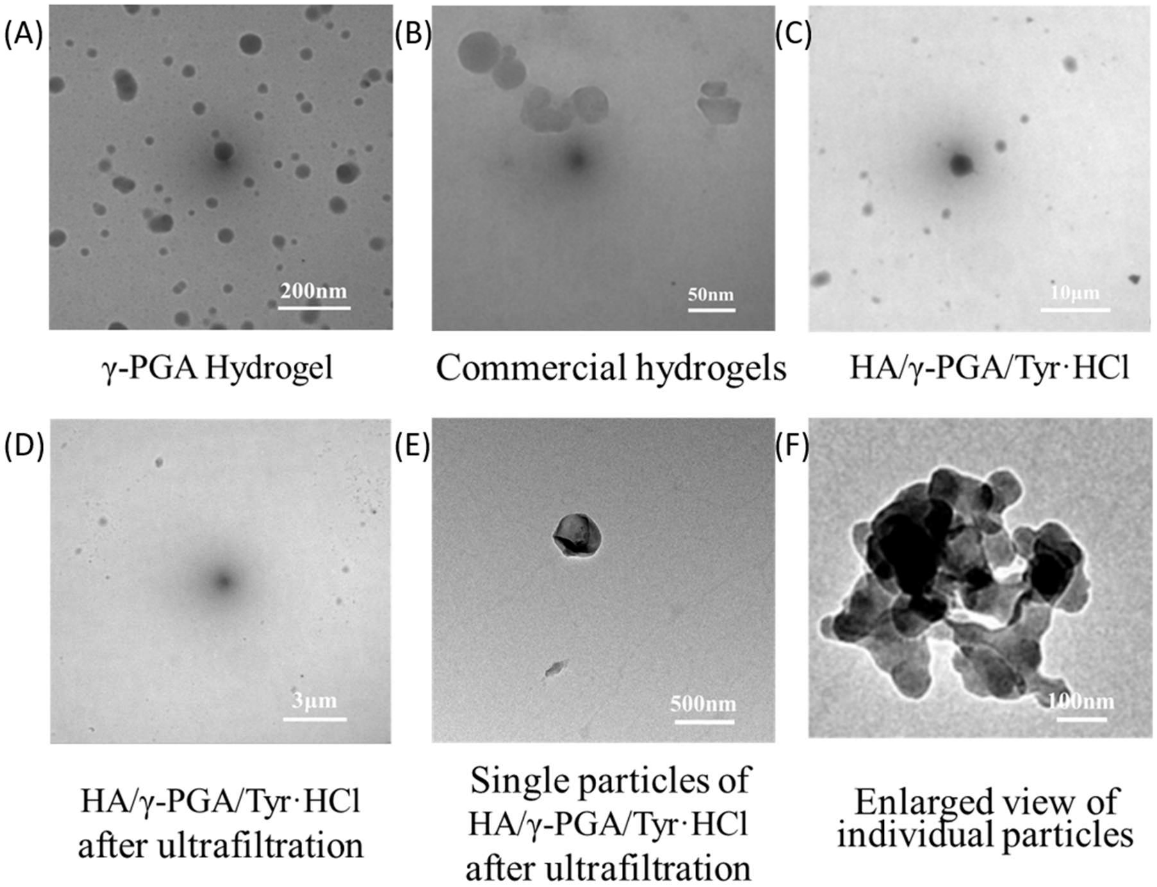

Figure S5. SEM images of different materials.

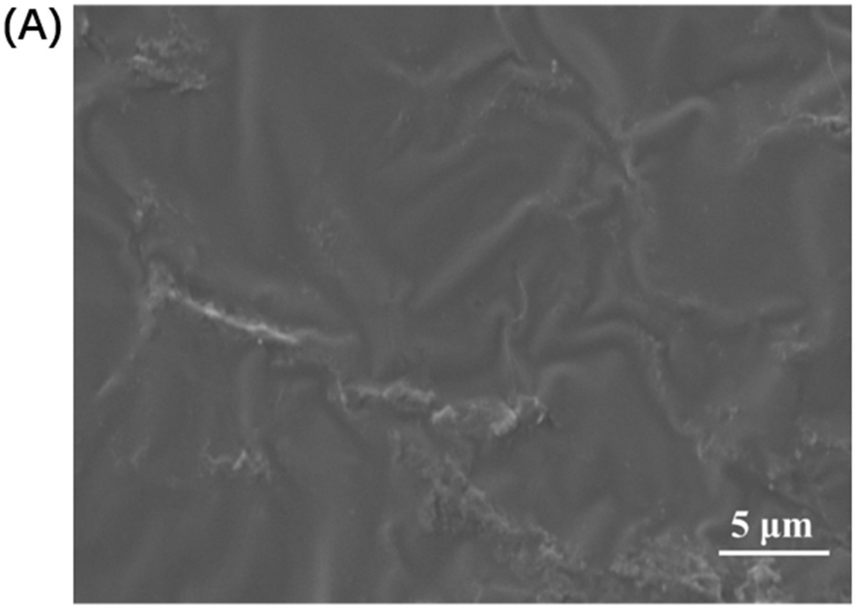

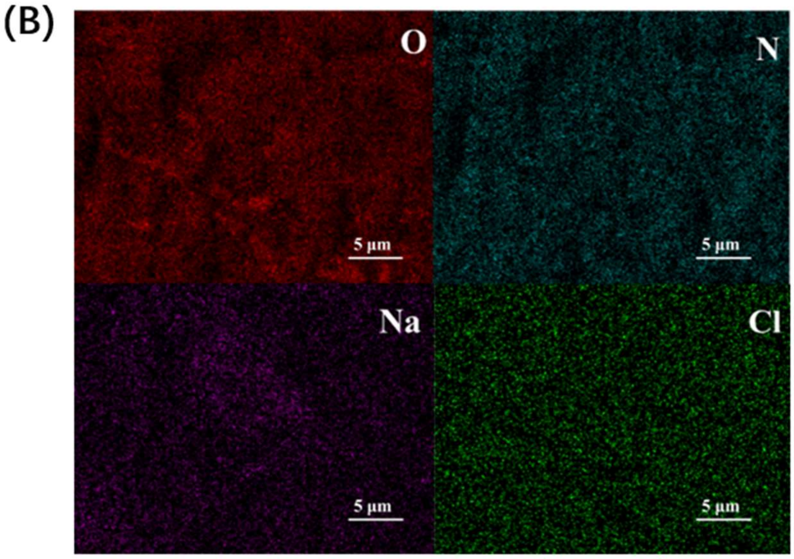

Figure S6. SEM(A) and mapping(B) of γ-PGA hydrogel precursor after ultrafiltration.

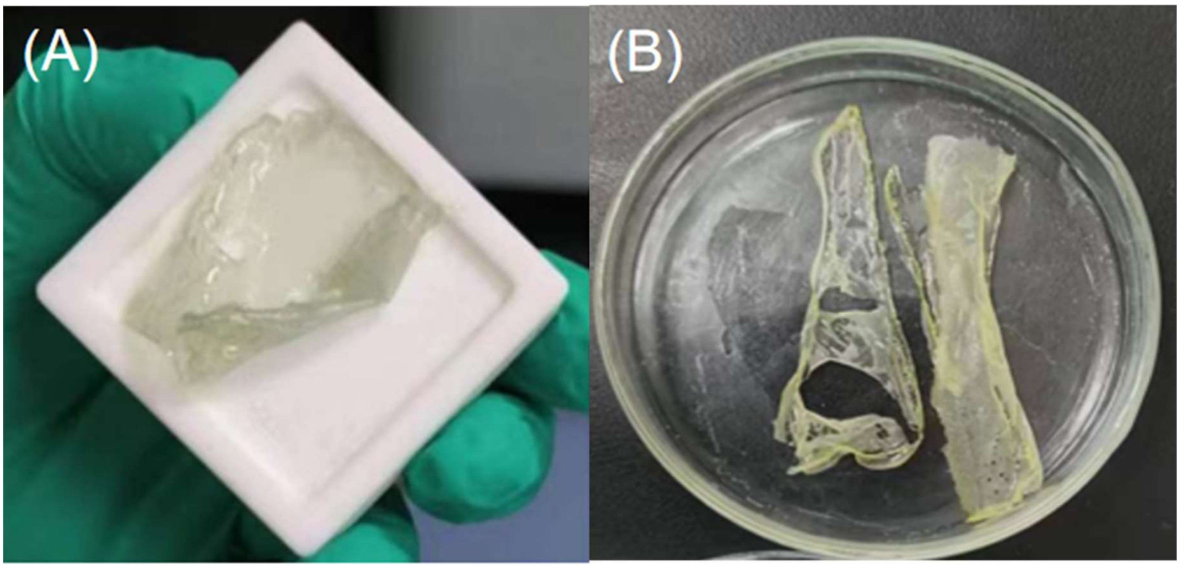

Figure S7. (A) Film-forming effect of dried γ-PGA micelles; (B) film-forming effect of Hi-Body brand sodium hyaluronate composite solution for injection.

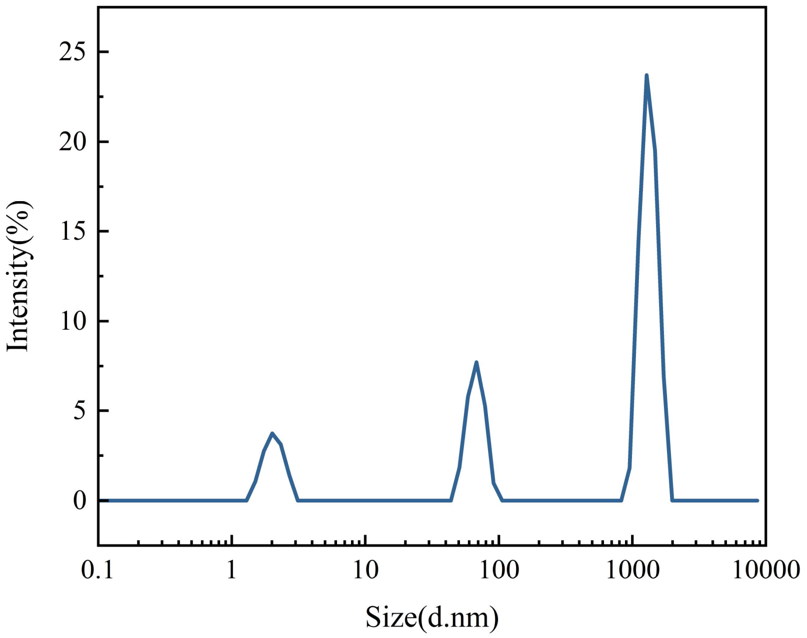

Figure S8. DLS image of commercial hyaluronic acid.

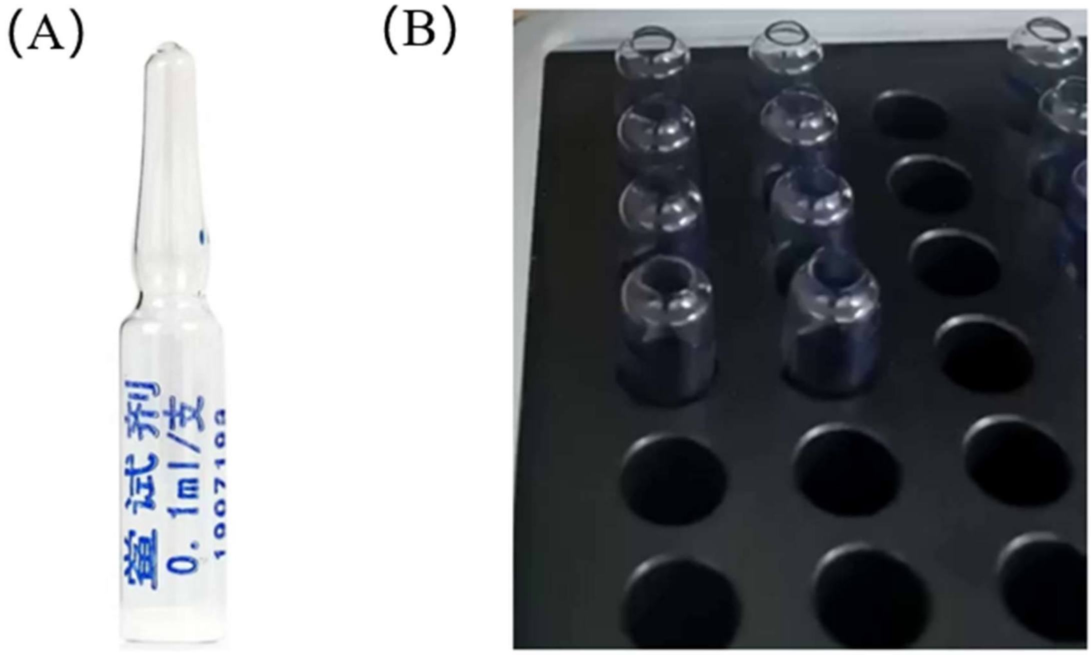

Figure S9. (A) Photograph of the horseshoe crab reagent; (B) photograph of the endotoxin analyzer in operation.

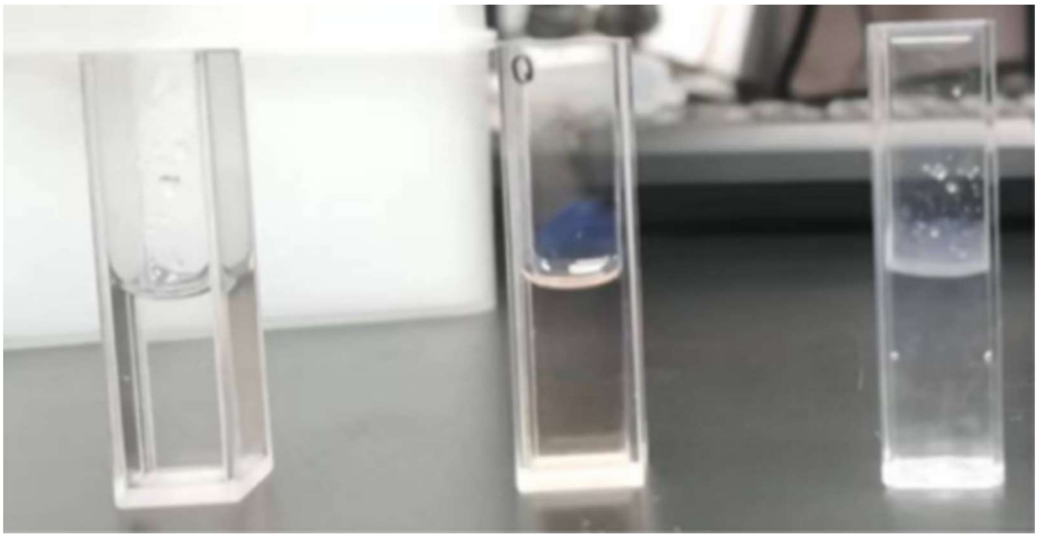

S10. Centrifuged Supernatant Physical Image.

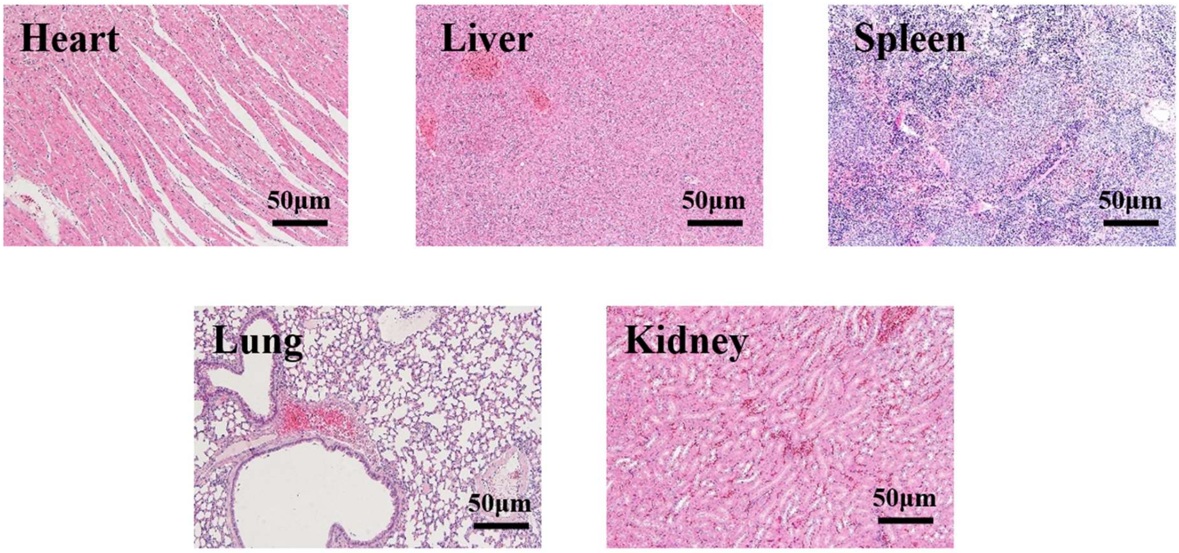

Figure S11. H&E staining of subcutaneously injected commercial injectable micelles in animal visceral tissue sections.

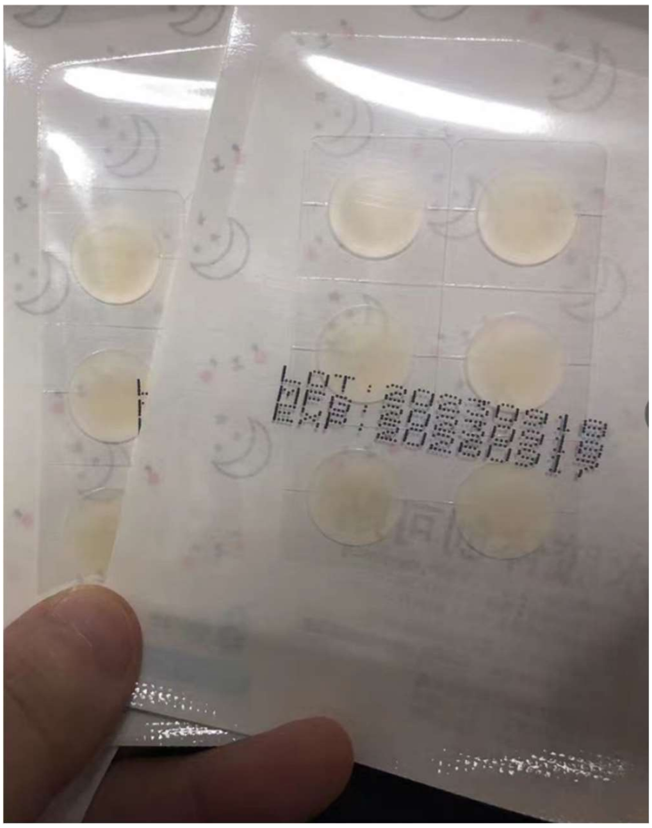

Figure S12. Actual image of a commercial hydrogel dressing.

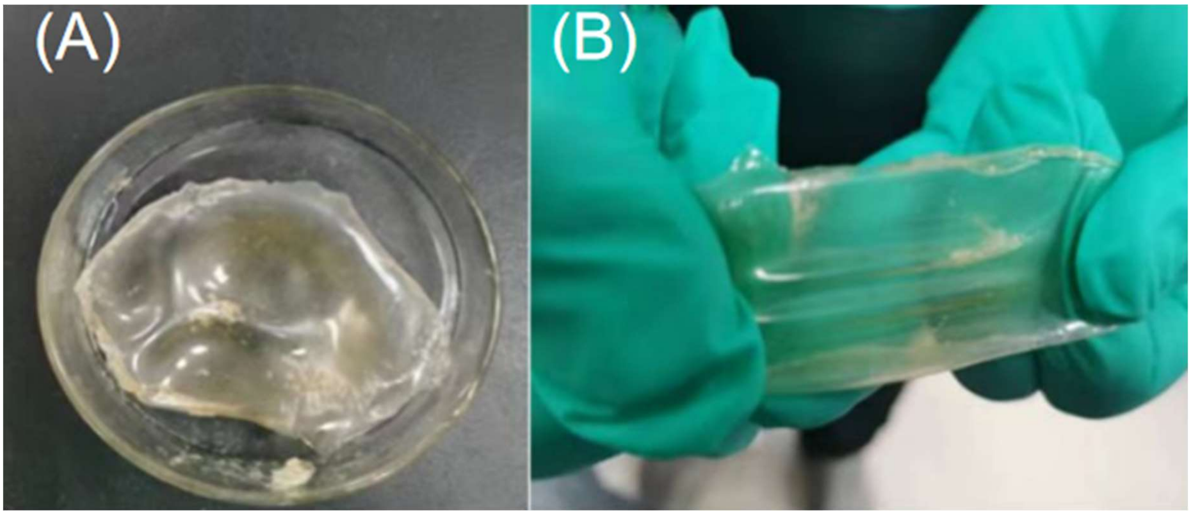

Figure S13 (A). Photograph of CS/γ-PGA/β-Ala hydrogel; (B) schematic diagram of manual stretching.

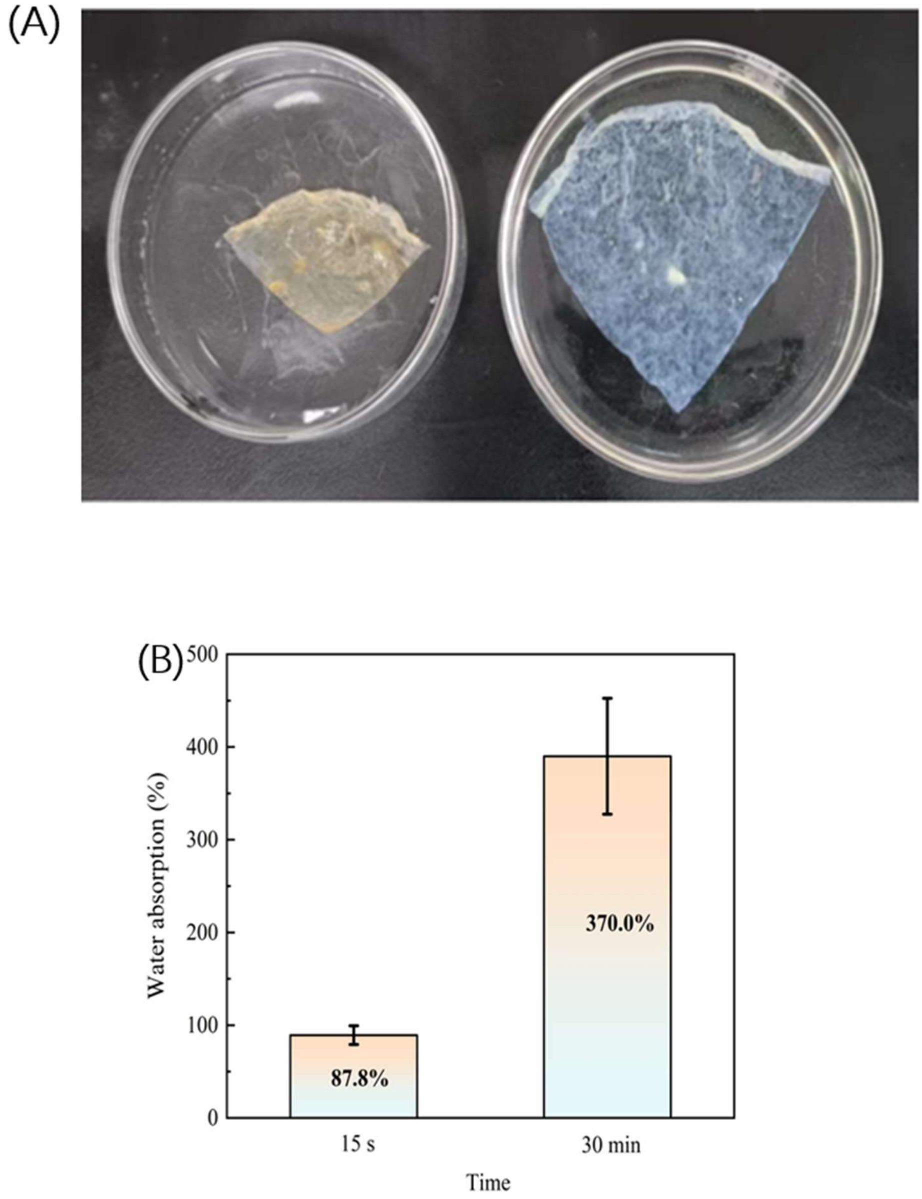

Figure S14. Actual image (A) and data chart (B) of water absorption capacity for γ-PGA hydrogel dressing ($p < 0.001$) (n=4).
